# Cloning and Transcriptional Activity Analysis of the Porcine Abcb1 Gene Promoter: Transcription Factor Sp1 Regulates the Expression of Porcine Abcb1

**DOI:** 10.3389/fphar.2018.00373

**Published:** 2018-05-04

**Authors:** Tingting Guo, Jinhu Huang, Changchao Huan, Fang He, Yujuan Zhang, Zohaib A. Bhutto, Liping Wang

**Affiliations:** ^1^Laboratory of Veterinary Pharmacology and Toxicology, College of Veterinary Medicine, Nanjing Agricultural University, Nanjing, China; ^2^Medical College, Yangzhou University, Yangzhou, China; ^3^College of Veterinary Medicine, Yangzhou University, Yangzhou, China

**Keywords:** Abcb1, promoter, transcription factor, regulation, Sp1, porcine

## Abstract

P-Glycoprotein (P-gp, Abcb1) plays a crucial role in drug disposition and functions by hydrolyzing ATP. However, little is known about the regulatory elements governing the transcription of the porcine *Abcb1* gene. In this study, the transcription start site of the pig *Abcb1* gene was identified by 5′-RACE. A 1.9-kb fragment of the 5′-flanking region of the *Abcb1* gene was cloned from pig genomic DNA and sequenced. The region critical for its promoter activity was investigated via progressive deletions. Further, using mutation assays, two proximal Sp1 binding sites within the 5′-flanking region of *Abcb1* were proven to be important *cis-*regulatory elements involved in regulating the constitutive expression of porcine *Abcb1*. RNA interference experiments showed that Sp1 regulated the expression of the porcine P-gp at both mRNA and protein levels. Hence, the current work provides valuable information on the regulatory mechanisms of pig Abcb1.

## Introduction

ABC transporters are a large superfamily of transmembrane proteins and are able to transport a broad range of substrates with preference for hydrophobic or cationic compounds by hydrolyzing ATP (Fletcher et al., 2010). P-Glycoprotein (P-gp), known as multidrug resistance protein 1 (MDR1) or ATP-binding cassette sub-family B member 1 (Abcb1), is one of the important ABC transporters because of its crucial role in drug absorption, distribution and excretion, so it draws significant interest in drug development. The expression level and activity of P-gp differentiate greatly between individuals due to genetic variations and environmental cues (Moriguchi et al., 2007). Despite an increasing interest in understanding the biological and pharmacological roles of P-gp in veterinary medicine (Zahner et al., 2010; Dunn et al., 2011; Yokota et al., 2011; Guo M. et al., 2016; Guo T. et al., 2016; Wilkens et al., 2016), little is known about how pig P-gp is regulated. Given the high expression of P-gp at numerous physiological barriers, it is of great importance to elucidate the molecular mechanisms of P-gp expression.

Promoters, which are usually located upstream of a gene, play a decisive role in gene expression. So far, nucleotide sequences of the *Abcb1* gene promoters have been identified from human, mouse, and hamster DNAs (Ueda et al., 1987; Raymond and Gros, 1990; Teeter et al., 1991). Molecular mechanisms for the transcriptional and post-transcriptional regulation of human P-gp have been extensively studied (Jin and Scotto, 1998). Previous studies have shown that several DNA elements and transcription factors are responsible for the basal or induced promoter activity (Goldsmith et al., 1993; Yasuda et al., 2015). It has been found that the human P-gp promoter does not contain a TATA promoter element, but a GC motif located at -56 to -42 of the human P-gp promoter is required for the constitutive promoter activity (Cornwell and Smith, 1993; Sundseth et al., 1997). Sp1, an important regulator that binds to GC-rich motifs, belongs to the SP/KLF transcription factor family (Hirose and Horvitz, 2013; Gonzalez-Ramirez et al., 2014). It exerts its role through binding to the promoter region of its target genes (Gazzoli and Kolodner, 2003), and can increase or decrease the transcription in response to physiological and pathological stimuli (Beishline and Azizkhan-Clifford, 2015).

Based on the important roles of the P-gp in swine drug disposition (Hsiu et al., 2002; Wang et al., 2004; Persson et al., 2008; Guo T. et al., 2016) and limited knowledge of the transcriptional regulatory mechanisms of the porcine *Abcb1* gene, we characterized the 5′-flanking region of the porcine *Abcb1* gene, and identified the core promoter region and *cis-*acting elements involved in the regulation of *Abcb1* expression. Our results indicate that the transcription factor Sp1 can bind to the proximal promoter and is required to regulate the expression of porcine *Abcb1* gene.

## Materials and Methods

### Animals

Animal studies were carried out according to the guidelines of the regional Animal Ethics Committee and the rules for experimental animals at Nanjing Agricultural University (Nanjing, China). Animal use and handling protocols were approved by the regional Animal Ethics Committee and Nanjing Agricultural University. Sixty-day-old healthy crossbred pigs (large white × Landrace × Duroc, 20 ± 2 kg) were purchased from Jiangsu Agricultural Academy (Nanjing, China) and reared under standard conditions of light (lights on, 07:00–21:00 h) and temperature (20–22°C). Jejunum from adult pigs were collected, snap-frozen in liquid nitrogen, and stored at -80°C until use.

### Rapid Amplification of 5′-cDNA Ends (5′-RACE)

**FIGURE 1:**
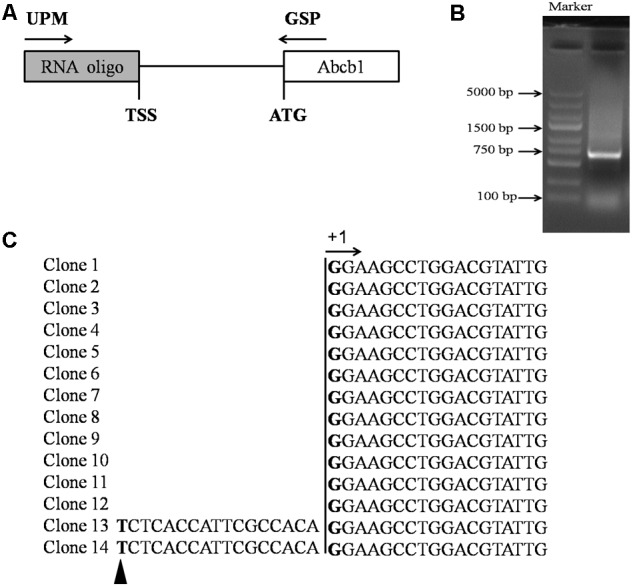
Identification of the TSS of porcine *Abcb1* gene. **(A)** Scheme of 5′-RACE. 5′-RACE PCR was performed with primer UPM (SMARTer^TM^ RACE 5′-primer) and primer GSP (gene-specific primer). **(B)** 5′-RACE PCR products were separated by gel electrophoresis. **(C)** Sequencing results of porcine Abcb1 5′-RACE clones are listed and the major TSS was defined as +1. Additional minor TSS position is marked with black triangle.

5′-RACE was performed using the SMARTer^TM^ RACE 5’/3’Kit (Clontech, Palo Alto, Japan) to identify the transcription start site (TSS) of pig *Abcb1*. Total RNA was extracted from the porcine jejunum tissue and reverse-transcribed using the reagent from the SMARTer^TM^ RACE 5′/3′Kit (Clontech, Palo Alto, Japan). The *Abcb1* gene-specific reverse primers (GSPs) were designed as shown in the diagram (**Figure 1A**) and are listed in **Table 1**. Reaction products were analyzed by agarose gel electrophoresis, then cloned into the pMD18-T vector (TaKaRa, Otsu, Japan) and sequenced.

**Table 1:** Oligonucleotide sequences of primers.

Name	Sequence (5′–3′)
**The pig *Abcb1* gene-specific primer for 5′-RACE PCR**	
GSP	CGATTCGGCCTTCTTCAAGATCCAT
**Primers for 5′ deletion constructs**	
pGL3-D1-F	GCATTGCTAGCTGCTAGAAACCTGTTAGAAA
pGL3-D2-F	GCATTGCTAGCAAAGAAATGCTAACAGTAAA
pGL3-D3-F	GCATTGCTAGCTTTTCTACTCGCGACACAAGG
pGL3-D4-F	GCATTGCTAGCCTAGTTGCTCTTTTGCTGAGGGGC
pGL3-D5-F	GCATTGCTAGCGCTCCTTCTAGGCCCCGAAGT
pGL3-deletion-R	GCATTAAGCTTACTCTCATTCCCCTGGCTCCT
**Primers used for qRT-PCR**	
Abcb1-F	AGTCTAATAAGAAGAGGAT
Abcb1-R	GCCATTCAGTTATATTCA
Gapdh-F	GAAGGTCGGAGTGAACGGAT
Gapdh-R	CATGGGTAGAATCATACTGGAACA
**Primers for site-directed mutagenesis**	
Sp1-mut1F	GCGGTCTGGCTGATTCTAGTTGCTCTAGAGCGCAG
Sp1-mut1R	CACCCCTGCGCTCTAGAGCAACTAGAATCAGCCAG
Sp1-mut2 F	TGGGCTGTAGAGCGCCTAGTTGCTCGCTGCACTTTTA
Sp1-mut2R	AGGAGTAAAAGTGCAGCGAGCAACTAGGCGCTCTACA
**Primers used for ChIP**	
Set A-F	GACATTCCTCCTGCAATTCCAAC
Set A-R	CTCAATACGTCCAGGCTTCCTGT
Set B-F	AGG AAG GGA CAG GAT GAG GA
Set B-R	TCA TGG TCT ATC CCA AGA GAC TG

*Underlined nucleotides were enzyme restriction sites.*

### Amplification of the 5′-Flanking Region of Pig *Abcb1* Gene

Genomic DNA, used for amplifying the 5′-flanking sequence of the pig *Abcb1* gene, was extracted from adult porcine jejunum using the Universal Genomic DNA Extraction Kit Ver. 5.0 (TaKaRa, Otsu, Japan). Based on the porcine genomic sequence GL880643.1, we designed the following primers to amplify the 5′-flanking region of pig *Abcb1*, 5′-TGC TAG AAA CCT GTT AGA AAG TCT AAA TAG CAG TTG-3′ (sense primer) and 5′-TTA TCC CTT TTC AAG AAG TTC TTC TTC CCT-3′ (antisense primer), generating a product of 1,905 bp from -1,843 nt to +62 nt (relative to the ATG initiation codon). Each 50 μl of PCR reaction mixture contained 10 μl of 5× PrimeSTAR GXL Buffer, 4 μl of dNTP Mixture, 1 μl of forward primer (10 μM), 1 μl of reverse primer (10 μM), 32 μl of water, 200 ng of genomic DNA, and 1 μl of PrimeSTAR GXL DNA Polymerase. The reaction conditions were as follows: 94°C for 5 min, then 32 cycles of 94°C for 30 s, 66°C for 30 s, and 72°C for 2 min, and finally at 72°C for 15 min. Amplicons were cloned using PMD18-T vector (TaKaRa, Otsu, Japan) and sequenced in both directions.

### Bioinformatics Analysis

The putative transcription factor binding sites were predicted using AliBaba2.1^1^ (Grabe, 2002) and the JASPAR database^2^ (Mathelier et al., 2016). The CpG islands were predicted by MethPrimer^3^ (Li and Dahiya, 2002).

### Construction of Reporter Plasmids

A series of plasmids containing fragments with various sizes of the 5′-flanking region of the porcine *Abcb1* gene were constructed in this study. All the primers used for the construction were tailed with Nhe I site (forward primers) or Hind III site (reverse primers) (**Table 1**). The amplified DNA fragments were digested with Nhe I and Hind III, and immediately inserted into the pGL3-basic vector (Promega, Madison, WI, United States) and sequenced. After sequencing verification, the plasmids (-1177-Luc, -777-Luc, -345-Luc, -195-Luc, +25-Luc) were extracted with an Endo-free Plasmid Mini Kit (Omega Bio-tek, Norcross, GA, United States) and quantified.

The Sp1 mutated vectors were constructed by the PCR-based site-directed mutagenesis using the Mut Express^®^ II Fast Mutagenesis kit (Vazyme, Nanjing, China) and Dpn? (Thermo, Rockford, IL, United States), following the manufacturer’s protocol. The -195-Luc vector containing the Sp1 binding sites was chosen as the template. The specific mutagenic primers are shown in **Table 1**. PCR products were amplified with the plasmids extracted from DMT competent cells (TransGen Biotech, Beijing, China) and confirmed by sequencing.

### Cell Culture and Transient Transfection Assay

IPEC-J2 (Intestinal porcine jejunal epithelial cell line J2) and Caco-2 cells were obtained from Shanghai Institute of Cell Biology, the Chinese Academy of Sciences (Shanghai, China) and grown in Dulbecco’s modified Eagle’s medium (DMEM, GIBCO, United States)/high glucose supplemented with 10% (v/v) fetal bovine serum (FBS, HyClone, Los Angeles, CA, United States), 100 unit/ml penicillin and 100 μg/ml streptomycin and maintained at 37°C in 5% CO_2_.

The cells were seeded in 24-well culture plates and cultured overnight, and then co-transfected with 0.7 μg of the reporter constructs and 0.05 μg of the Renilla luciferase reporter plasmid pRL-TK (Promega, Madison, WI, United States) to normalize for transfection efficiency using Lipofectamine^TM^ 2000 Reagent (Invitrogen, Carlsbad, CA, United States). The luciferase activity was determined 24 h after the transfection using a Dual-luciferase^®^ Reporter Assay Kit (TransGen Biotech, Beijing, China) and a dual luciferase reporter assay system (Promega, Madison, WI, United States). A promoter-null plasmid, pGL3-Basic, was also included in the transfection assay as a negative control (NC). All transfections were carried out in triplicates and repeated at least in three independent experiments.

### RNA Interference

Silence efficiency of the small interfering RNAs (siRNAs) was detected first. Briefly, cells were seeded in 6-well culture plates and grown to 60–70% confluence. Then the cells were transiently transfected with 50 nM siRNA for 36 h using Lipofectamine^TM^ 2000 reagent according to the manufacturer’s instructions. After transfection, the cells were collected to detect the expression of Sp1 by western blotting as described previously (Guo T. et al., 2016). At the same time, the collected cells were used to detect the expression of P-gp expression by qRT-PCR and Western blotting. siRNAs targeted to the fragment “CCTGGAGTGATGCCTAATA” were prepared by Biotend (Shanghai, China), and used to silence the expression of the porcine Sp1. The sequences of the Sp1 siRNAs and the primers used in qRT-PCR are shown in **Table 1**. Western blotting experiments were done with primary antibody at the appropriate dilution (Sp1, Santa Cruz, Germany, 1:500; Mdr-1, Santa Cruz, Germany, 1:200; β-actin, TransGen, China, 1:5,000). The membranes were washed three times with PBS-T before incubated with the appropriate secondary antibody (HRP-anti-rabbit IgG or HRP-anti-mouse IgG) for 1 h. The immunoreactive bands were visualized by an ECL enhanced chemiluminescence system (Vazyme, Nanjing, China). After proving the Sp1 siRNA with a good interference efficiency, pGL3-D4 construct (0.7 μg), the Renilla luciferase reporter plasmid pRL-TK (0.05 μg) and 12 nM siRNAs were co-transfected into the cells with 60–70% confluence in 24-well culture plates for 36 h using Lipofectamine^TM^ 2000 reagent according to the manufacturer’s instructions. The luciferase activity was determined as described above.

### Chromatin Immunoprecipitation (ChIP) Assay

The ChIP assay was conducted following the instruction of the ChIP assay Kit (Beyotime, Shanghai, China). The IPEC-J2 cells cultured in 100 mm dishes were fixed and neutralized. Briefly, proteins were cross-linked to DNA with 1% formaldehyde for 10 min at room temperature. Then, the reaction was quenched by adding glycine (a final concentration of 125 mM) to the media with shaking for 5 min and washed three times with PBS. The cells were scraped with PBS, and centrifuged at 2000 × *g* for 4 min at 4°C. Then the cells were lysed with 25°C SDS lysis buffer for 10 min on ice. After washing with cold PBS, the cells were harvested, and then sonicated to produce chromatin fragments about 200–750 bp in size. Fragmented chromatin was added into ChIP dilution buffer, and incubated overnight with anti-Sp1 (H-225X, Rabbit polyclonal antibody, Santa Cruz, Germany). A Normal Rabbit IgG (Cell Signaling, Beverly, MA, United States) was added as a NC. Immunoprecipitated products were collected after incubation with Protein A + G coated magnetic beads. The bound chromatin was eluted and digested with proteinase K, and the DNA was then purified for PCR analysis (the primers set A and B are listed in **Table 1**). Primers set B were designed toward 2 kb upstream of the promoter, which serves as a control for the specificity.

### Statistical Analysis

All experiments were performed in triplicates and repeated at least in three independent experiments. Data were presented as mean ± standard deviation (SD) of three replications. Mean values between groups were compared using the two-tailed Student’s *t*-test. Differences were considered significant at *p* < 0.05.

## Results

### Identification of the Transcription Start Site (TSS) of Porcine *Abcb1* Gene

To better understand the transcriptional regulation of porcine *Abcb1* gene, we first determined the exact TSS of *Abcb1* by 5′-RACE. Our previous study indicated that P-gp was highly expressed in the jejunum of pigs (Guo T. et al., 2016), therefore, the jejunum was selected to extract the RNA as the template for 5′-RACE to determine the TSS of *Abcb1*. The PCR products with the size of 700 bp were obtained using GSP (**Figure 1B**). After cloning and sequencing of 14 different clones, we identified the major *Abcb1* TSS (**Figure 1C**, +1) located at 666 bp upstream of the ATG. Only two RACE clones showed a slightly different TSS (**Figure 1C**, black triangle), and this site was identified as an additional minor TSS, which often occurred in TATA-less promoter. The major TSS was defined as +1 for later illustration.

### Discovery of Several Transcription Factor Binding Sites in Pig Abcb1 Promoter

**FIGURE 2:**
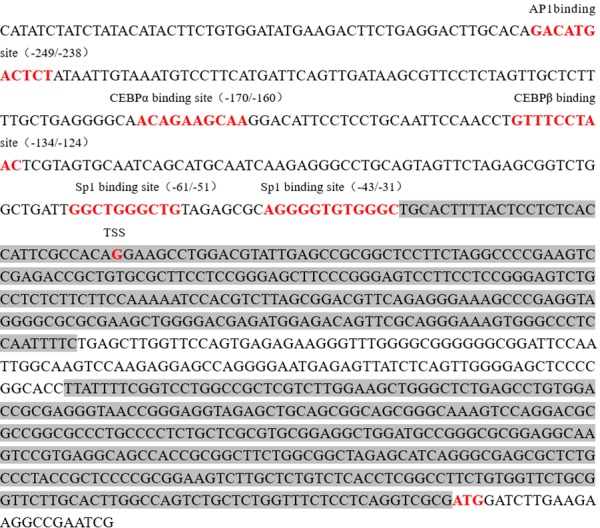
Nucleotide sequence of the promoter region (–303 to +691) of porcine *Abcb1* gene. The TSS was designated as +1. The putative binding sites for the transcription factors were predicted using AliBaba2.1 and JASPAR database and marked in red on the sequence. CpG islands were predicted by MethPrimer and marked with gray background.

A total of 1, 905 bp 5′-flanking sequence of *Abcb1* gene was obtained from the pig genomic DNA. Several transcription factor binding sites, including AP1, C/EBP α, C/EBP β and Sp1 binding sites, were recognized in this region by analyzing the 5′-flanking sequences (**Figure 2**). A putative binding site was found in bases -249 to -238, which was usually recognized by AP1. The binding sites for C/EBP α and C/EBP β were found in bases -170 to -160 and -134 to -124, respectively (**Figure 2**). Two putative Sp1 binding sites were also found in the bases -61 to -51 and -43 to -31, respectively (**Figure 2**). In addition, two CpG islands were predicted in the pig *Abcb1* promoter using MethPrimer (**Figure 2**), indicating that the methylation level of the CpG islands might influence the transcription of pig *Abcb1*.

### Characterization of *Abcb1* Gene Core Promoter

**FIGURE 3:**
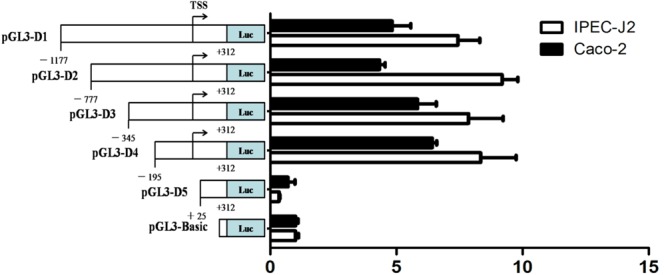
5′-Deletion analysis of the porcine Abcb1 promoter activity. Five promoter constructs were transfected into IPEC-J2 and Caco-2 cells, and assayed for luciferase activity. Schematic diagrams of truncated promoters **(left)**. The corresponding luciferase reporter assay results **(right)**. Results were presented as normalized relative luciferase activity (Firefly/Renilla). Data were expressed as means ± SD of three replicates.

To investigate the core promoter of porcine *Abcb1* gene, we generated a series of deletion luciferase reporter constructs of the 5--region of the gene (**Figure 3**, left), which were transiently transfected into the pig-derived IPEC-J2 cells and human-derived Caco-2 cells, respectively. The luciferase activity derived from *Abcb1* promoter with different sizes was determined using Dual-Luciferase^®^ Reporter Assay System. As shown in **Figure 3**, transfection of (-1177∼+312)-Luc construct into IPEC-J2 cells resulted in an eightfold induction of luciferase activity compared with the pGL3-Basic vector (*p* < 0.05). Further deletions to bases -777, -345, and -195, respectively, did not cause significant difference of the luciferase activities compared with each other, indicating that there are no important regulatory elements within these regions (**Figure 3**). We noticed that the luciferase activity was not diminished by deleting DNA up to position -195 (pGL-195/+312). However, the removal of the region (-195 to +24) remarkably decreased the luciferase activity of the (+25 to +312)-luc construct to 4.2% of the (-195 to +312)-luc construct, strongly suggesting that there are *cis*-acting elements responsible for the constitutive expression of *Abcb1* in the region from bases -195 to +24 (**Figure 3**).

### Sp1 Regulation of the Constitutive Expression of Porcine *Abcb1*

**FIGURE 4:**
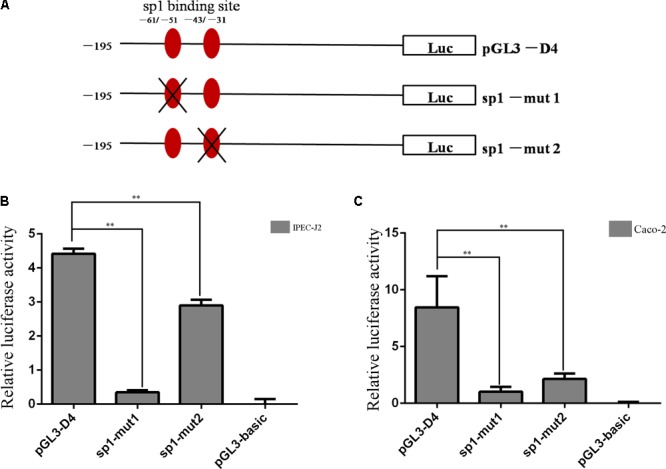
Site-directed mutation of Sp1 binding sites. **(A)** Schematic structure of site-directed mutagenesis in the putative Sp1 binding sites of porcine Abcb1 promoter. **(B,C)** Luciferase activity of site-directed mutagenesis in IPEC-J2 and Caco-2 cells, respectively. Statistical differences of relative activities were analyzed in the same cells; ^∗∗^*p* < 0.01, data were expressed as means ± SD of three replicates.

As shown in **Figure 2**, two Sp1 binding sites were located in this region (-195 to +24). Therefore, to further functionally determine the contribution of Sp1 binding sites to the constitutive expression of porcine *Abcb1*, site-directed mutagenesis, which can disturb the recruitment of the transcription factors, was performed (**Figure 4A**). The newly generated constructs (with -61/-51 and -43/-31 mutations) were transfected into IPEC-J2 and Caco-2 cells, respectively. Luciferase activities were measured with the pGL3-D4, Sp1-mut1, and Sp1-mut2 in both IPEC-J2 and Caco-2 cells (**Figures 4B,C**), respectively. Their effects on promoter activities were examined by dual luciferase assay. As shown in **Figure 4B**, the mutation in -61/-51 region significantly reduced the relative luciferase activity in IPEC-J2 cells to about 8% of the wild-type control (pGL3-D4), whereas the mutation in -43/-31 region decreased the luciferase activity to 66% of the wild-type control (pGL3-D4), indicating that the promoter activity of *Abcb1* is more significantly inhibited by the dysfunction of -61/-51 region than -43/-31 region. Similar results were observed in Caco-2 cells where the mutation in -61/-51 region had more significant effect on the relative luciferase activity than the mutation in -43/-31 region (**Figure 4C**), but the mutation in -43/-31 region led to quantitative difference in the relative luciferase activity in the two cell lines.

**FIGURE 5:**
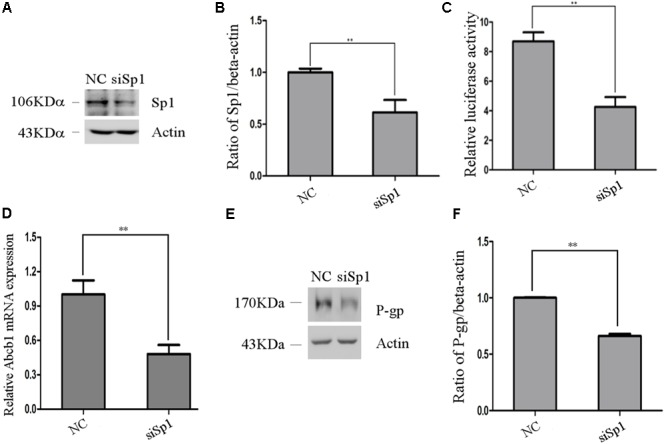
The effect of Sp1 knockdown on pig Abcb1 promoter activity and endogenous expression of pig P-gp expression in IPEC-J2 cells. **(A)** Downregulation of Sp1 protein level by specific siRNAs in IPEC-J2 cells was confirmed by Western blot, as compared to the negative control (NC) siRNA. **(B)** Semi-quantification of Western blot. **(C)** IPEC-J2 cells were co-transfected with pGL3-D4 construct and Sp1 siRNA or NC siRNA for luciferase assays. The Renilla luciferase reporter plasmid pRL-TK (Promega) was used as an internal control to estimate the transfection efficiency. ^∗∗^*p* < 0.01. **(D)** The effect of inhibition of Sp1 on *Abcb1* mRNA expression in IPEC-J2 cells was analyzed by qRT-PCR. ^∗∗^*p* < 0.01. **(E)** The effect of inhibition of Sp1 on P-gp protein expression in IPEC-J2 cells was analyzed by Western blot. **(F)** Semi-quantification of P-gp expression.

To further verify the role of Sp1 in the regulation of porcine Abcb1 promoter activity, western blot was performed to detect the efficiency of siRNA-Sp1 (siSp1). The results are shown in **Figures 5A,B**. Then, we co-transfected the reporter plasmid pGL3-D4 together with siRNA-Sp1 (siSp1) or NC into IPEC-J2 cells individually. As shown in **Figure 5C**, when Sp1 was silenced by specific siRNAs, porcine Abcb1 promoter activity decreased by about 50% compared to NC. Accordingly, the expression levels of Abcb1 were effectively reduced by siRNA-Sp1 in IPEC-J2 cells, as detected by qRT-PCR and western blot (**Figures 5D–F**). Taken together, the data presented here confirmed that Sp1 acted as a positive regulator of *Abcb1* transcription.

### Sp1 Binding to the Porcine Abcb1 Promoter

**FIGURE 6:**
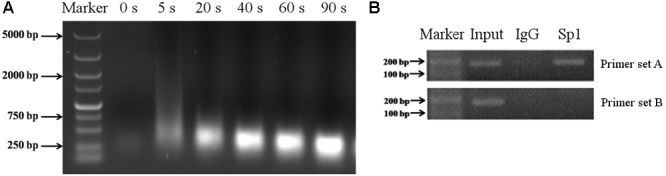
The results of ChIP experiment. **(A)** The optimized sonication time for ChIP assay was explored. **(B)** ChIP assay of Sp1 binding to porcine Abcb1 promoter in IPEC-J2 cells. DNA isolated from immunoprecipitated materials was used for PCR amplification; Normal rabbit IgG was used as NC; total chromatin was used as input (positive control).

To determine whether Sp1 binds to the promoter region of porcine *Abcb1*, ChIP analysis was performed in IPEC-J2 cells. For ChIP assay, the genomic DNA should be sheared uniformly and randomly, and the DNA fragment size after sonication should be 200–750 bp (Nelson et al., 2006; Walton and Matter, 2015). Thus, choosing the best sonication time is a critical step. In this study, we found that the most optimized sonication time was 40 s (**Figure 6A**). With the particular set of primers set A (**Table 1**), bands were detected in the input lane (**Figure 6B**). Besides, the bands with the expected size were also observed when anti-Sp1 antibody was added (**Figure 6B**), indicating that Sp1 could interact with the Abcb1 promoter. Conversely, no DNA band was amplified from the normal rabbit IgG immunoprecipitated chromatin with the primers set A (**Figure 6B**). With the primers set B, bands were detected in the input lane but not in the immunoprecipitated sample lane. These findings suggested that the proximal Sp1 binding sites of the Abcb1 promoter were able to bind to Sp1 protein.

## Discussion

Previous studies have suggested that human and rodent *Abcb1* genes are regulated by different mechanisms (Burt and Thorgeirsson, 1988; Chin et al., 1990). By analyzing the promoter of pig *Abcb1* gene, we found that a GC rich region was located in the promoter, which was highly conserved in ABC transporter genes (Cornwell and Smith, 1993). However, by comparing the whole promoter sequences of porcine Abcb1 with those of human and mouse, it shown that there is very low similarity. Thus, it is necessary to identify the core region of the promotor first. In light of lacking eukaryotic promoter and enhancer sequences in pGL3-basic vector, the sequences inserted upstream from luc^+^ affect the expression of the luciferase activity in cells (Tang et al., 2015). Using this vector, we found that the 5′-flanking region from -195 to +25 bp of porcine *Abcb1* gene significantly influenced the promoter activity (**Figure 3**), suggesting that the core promoter is located in this region and the regulatory elements of this region may affect the promoter activity of porcine Abcb1. Notably, the promoter activity of the pGL3-D5 was even lower than that of the pGL3-basic vector. This might be due to the presence of a short open reading frame (sORF) in the pig Abcb1 promoter, which sometimes occurs in the promoter of other gene, like pig *ADRB2* (Jaeger et al., 2015). To the best of our knowledge, this study, for the first time, comprehensively described the characterization of the 5′-flanking region of porcine *Abcb1* and the molecular mechanisms that regulates the constitutive expression of porcine *Abcb1*.

By using JASPAR and AliBaba2.1, two potential binding sites for Sp1 were predicted in the core promoter regions of porcine *Abcb1*. It has been described that Sp1 positively regulates the expression of the rat *Abcb1b* gene (Thottassery et al., 1999), and the multiple Sp1 sites in the human Abcb1 promoter are not equally functioning (Sundseth et al., 1997). Based on the inequality roles of Sp1 sites, different Sp1 mutation plasmids were constructed. Site mutation results suggested that the mutations of Sp1 binding site at position from -61 to -51 and position from -43 to -31 significantly reduced the transcriptional activities of Abcb1 promoter by 92 and 34% in IPEC-J2 cells, respectively (**Figure 4**). However, when we transfected the mutant plasmids into Caco-2 cells, the mutations of Sp1 binding site at position from -61 to -51 and position from -43 to -31 significantly reduced the transcriptional activities of Abcb1 promoter by 88 and 75%, respectively (**Figure 4**). The results indicated that Sp1 binding sites were *cis-*regulating motif in the porcine Abcb1 promoter, which was the same in the human Abcb1 promoter proved in the *Drosophila* cells (Cornwell and Smith, 1993). Additionally, the ChIP assay revealed that Sp1 did bind to the Abcb1 promoter directly and these interactions were important determinants of the basal promoter activity. Taken together, Sp1 acts as a critical regulatory factor for porcine *Abcb1* transcription and thus affects the bioavailability or drug–drug interaction of substrate xenobiotics.

Understanding whether a drug is an inducer or inhibitor of pig Abcb1 is also important for predicting drug–drug interactions in veterinary medicine and may be a strategy for developing new drugs. The current Food and Drug Administration guidance on evaluating P-gp induction potential of a new chemical entity is not based on direct evaluation of whether a drug induces P-gp, but depends on whether it induces CYP3A (Zhang et al., 2009). Based on the tissue and species differences in induction of CYP3A and Abcb1 (Schuetz et al., 1996; Hartley et al., 2004), we propose that detecting the effect of the drug on pig Abcb1 promoter may be a good approach to identify P-gp inducers or inhibitors.

In summary, here we, for the first time, identified and characterized the TSSs and core promoter of the pig Abcb1 gene. Our results demonstrated that Sp1 could bind to the Abcb1 promoter directly to regulate the expression of the porcine P-gp at both mRNA and protein levels.

## Author Contributions

TG, CH, and LW: participated in the research design. TG, JH, and CH: conducted the experiments. TG, FH, YZ, and ZB: performed the data analysis. TG and LW: wrote or contributed to the writing of the manuscript.

## Conflict of Interest Statement

The authors declare that the research was conducted in the absence of any commercial or financial relationships that could be construed as a potential conflict of interest.

## Footnotes

1 http://www.gene-regulation.com/pub/programs.html

2 http://jaspar.genereg.net/

3 http://www.urogene.org/methprimer/
